# LMT2368 (1-(4-Chlorophenyl)-3-(3-fluoro-5-(trifluoromethyl)phenyl)urea) Negatively Regulates Inflammation by Inhibiting NLRP3 Inflammasome Activation

**DOI:** 10.3390/pharmaceutics17101241

**Published:** 2025-09-23

**Authors:** Thai Uy Nguyen, Su Jeong Kwon, Sunghoon Hurh, Ashok Kale, Jae Min Cho, Hossam Nada, Chang Seong Kim, Peela Induvadana, Beom Jin Park, Kyeong Lee, Yongseok Choi, Jong-Ik Hwang

**Affiliations:** 1 Department of Biomedical Science, College of Medicine, Korea University, Seoul 73 Goryeodae-ro, Seongbuk-gu, Seoul 02841, Republic of Korea; nguyenthaiuy@gmail.com (T.U.N.); shurh@korea.ac.kr (S.H.); 2 Brain Korea 21 Fostering Outstanding Universities for Research, Team and Integrated Research Institute for Drug Development, College of Pharmacy, Dongguk University, Seoul 02841, Republic of Korea; plus2304@naver.com (S.J.K.); ashokkale9065@gmail.com (A.K.); whwoals15@dgu.ac.kr (J.M.C.); hossam_hammouda@dgu.ac.kr (H.N.); kcs980523@gmail.com (C.S.K.); 3 Department of Radiology, Molecular Imaging Innovations Institute (MI3), Weill Cornell Medicine, New York, NY 10065, USA; 4 College of Life Sciences and Biotechnology, Korea University, Seoul 02841, Republic of Korea; induvadana1@gmail.com (P.I.); ychoi@korea.ac.kr (Y.C.); 5 Department of Radiology, College of Medicine Anam Hospital, Korea University, 73 Goryeodae-ro, Seongbuk-gu, Seoul 02841, Republic of Korea; bjp226@korea.ac.kr

**Keywords:** LMT2368, NLRP3, inflammasome, IL-1β, acute lung injury

## Abstract

**Background/Objectives**: The dysregulation of NLRP3 inflammasome activation has been established as a key driver of inflammatory disease pathology, which marks NLRP3 as an attractive therapeutic target. However, the clinical development of NLRP3 inhibitors such as MCC950 has been hampered by their associated toxicity profiles, highlighting an unmet clinical need. **Methods**: Herein, we present LMT2368, a novel urea-based NLRP3 inhibitor identified through screening of urea-based derivatives from our in-house compound library. **Results**: Biolayer interferometry confirmed direct binding of LMT2368 to the NLRP3 NACHT domain with a (*K*_D_ = 27.4 ± 1.2 μM which was superior to MCC950. Molecular docking studies predicted enhanced binding interactions for LMT2368, consistent with its improved biological activity. In LPS-primed macrophages, LMT2368 dose-dependently suppressed IL-1β secretion (IC50 = 0.8 μM in J774A.1 cells) and caspase-1 activation without affecting NF-κB signaling. Importantly, LMT2368 inhibited ASC oligomerization and pyroptosis while maintaining excellent safety margins (CC50 > 50 μM). In a murine model of LPS-induced acute lung injury, LMT2368 (10 mg/kg) reduced bronchoalveolar lavage fluid immune cell infiltration by 68% (*p* < 0.001), suppressed pro-inflammatory cytokine release (IL-1β/IL-6/TNF-α), and preserved lung histoarchitecture. Notably, LMT2368 showed selectivity for NLRP3 inhibition without affecting TNF-α/IL-6 production during TLR4 priming in monocytic cell lines. **Conclusions**: Together, these findings establish LMT2368 as a promising lead compound for developing safer NLRP3 inhibitors with therapeutic potential for inflammasome-driven diseases.

## 1. Introduction

Innate immunity serves as the primary line of defense against inflammatory stimuli triggered by both exogenous and endogenous pathogenic factors, collectively termed pathogen-associated molecular patterns (PAMPs) and damage-associated molecular patterns (DAMPs). PAMPs are derived from microbes, while DAMPs originate from trauma, chemically induced damage, or dead cells [1,2,3]. These stimuli are recognized by pattern recognition receptors (PRRs) located on the cell membrane or within the cytosol. Among these, Toll-like receptors (TLRs) are well-characterized PRRs that detect conserved microbial components such as lipopolysaccharide (LPS) from Gram-negative bacteria, bacterial or viral nucleic acids, bacterial peptides like flagellin, and polysaccharides, including β-glucans [4,5,6]. Activation of these receptors initiates signaling cascades that culminate in the formation of multimeric protein complexes known as inflammasomes [7], which play a crucial role in the expression, activation, and release of pro-inflammatory cytokines, thereby propagating inflammatory signals both locally and systemically [7,8].

Recent studies have elucidated critical upstream regulators of inflammasome activity. The intracellular proteasome, which mediates selective degradation of ubiquitin-tagged proteins, plays a pivotal role in controlling the abundance and turnover of key inflammatory mediators, including IκB [9]. Proteasome-dependent IκB degradation is necessary for NF-κB activation, which primes the transcriptional program required for NLRP3 expression and full inflammasome assembly [9]. Furthermore, mitogen-activated protein kinases (MAPKs)—notably p38 and JNK—link extracellular stress to nuclear transcriptional responses, enhancing NF-κB/AP-1-driven expression of NLRP3 and pro-IL-1β during both infectious and sterile inflammation [10].

Over the past two decades, the molecular composition and activation mechanisms of various inflammasomes have been extensively characterized [11,12]. Among these, the NOD-like receptor family pyrin domain-containing 3 (NLRP3) inflammasome is distinguished by its responsiveness to diverse pathogenic and damage-associated stimuli and its involvement in numerous inflammatory diseases [13,14,15]. The NLRP3 inflammasome is composed of NLRP3 (the sensor), apoptosis-associated speck-like protein containing a caspase-recruitment domain (ASC, the adaptor), and pro-caspase-1 (the effector). NLRP3 contains three distinct domains: a C-terminal leucine-rich repeat (LRR) involved in stimulus recognition; a central NACHT domain responsible for oligomerization; and an N-terminal pyrin domain (PYD) that recruits ASC, facilitating inflammasome assembly [16,17].

Activation of the NLRP3 inflammasome occurs via both canonical and non-canonical pathways [18,19]. Canonical activation entails a two-step process: a priming, where PAMPs or DAMPs induce NF-κB-dependent upregulation of NLRP3, pro-IL-1β, and pro-IL-18; and activation, where stimuli such as ATP, toxins, or pathogens trigger structural rearrangement, NEK7 binding, and subsequent protein complex assembly [20]. ASC then enables pro-caspase-1 recruitment and activation to drive cytokine maturation and pyroptosis, which releases pro-inflammatory mediators into the extracellular space [21,22]

Although NLRP3 inflammasome activation is essential for host defense and tissue repair, its dysregulation precipitates a spectrum of acute and chronic inflammatory diseases, including autoimmunity, metabolic syndromes, and respiratory pathologies [15,23]. As such, NLRP3 has emerged as an attractive therapeutic target, leading to extensive drug discovery efforts around small-molecule NLRP3 inhibitors such as MCC950. However, MCC950′s clinical development has been limited by hepatotoxicity, off-target effects, and pharmacokinetic liabilities, revealing an unmet clinical need for potent and safer NLRP3 inhibitors [24,25,26,27].

In this study, we conducted a pharmacological screening of urea-containing scaffolds from our in-house library for their ability to inhibit NLRP3 inflammasome activation. Among the candidates, LMT2368 emerged as the most effective compound. LMT2368 was shown to directly interact with NLRP3, potentially impeding inflammasome assembly and, consequently, preventing caspase-1 activation in monocytic cells and primary peritoneal macrophages. We further assessed its effects in a murine model of LPS-induced acute lung injury. Our findings demonstrate that LMT2368 significantly reduces lung inflammation by decreasing the expression of key inflammatory markers and modulating immune cell infiltration, establishing LMT2368 as a promising lead for future development as an NLRP3-targeted therapeutic.

## 2. Materials and Methods

### 2.1. Reagents and Antibodies

LPS (*Escherichia coli* 0111:B4) was purchased from Sigma-Aldrich (St. Louis, MO, USA). Cell culture media were from WELGENE Inc. (Daegu, Korea). The protease inhibitor cocktail was from Roche (Mannheim, Germany). Antibodies targeting p-Akt, Akt, p-NF-κB p65, NF-κB p65, p-STAT3, and p-ERK1/2 were from Cell Signaling Technology (Beverly, MA, USA). Anti-β-actin was from Santa Cruz Biotechnology (Santa Cruz, CA, USA). Anti-ASC antibodies (Cat. No. ab309497) were purchased from Abcam (Cambridge, UK). Anti-Caspase-1 (p20) antibodies (Cat. No. AG-20B-0042-C100) were purchased from AdipoGen Life Science (San Diego, CA, USA). Ly6G/6C (#14-5931-82), F4/80 (#14-4801-82), and Anti-IgG Alexa Fluor 488 (#A21208) antibodies were purchased from Invitrogen (Thermo Fisher Scientific, Waltham, MA, USA). PE-Anti CD31 (#555027) antibody was purchased from BD Pharmingen (BD Biosciences, San Jose, CA, USA). Enzyme-linked immunosorbent assay (ELISA) kits for mouse interleukin-1β (IL-1β), interleukin-6 (IL-6), and tumor necrosis factor-α (TNF-α) were from Invitrogen (Carlsbad, CA, USA). Primers for RT-PCR and QRT-PCR were from Cosmo Genetech (Seoul, Korea). All other reagents were from Sigma-Aldrich (St. Louis, MO, USA) unless otherwise stated. The synthesized chemicals used for the NLRP3 inhibition assay are described in the Supplementary Materials.

### 2.2. Cell Culture

J774A.1 cells and U937 cells (J774A.1 and U937 cells are from American Type Culture Collection (ATCC, Gaithersburg, MD, USA)) were cultured in RPMI-1640 medium supplemented with 10% (*v*/*v*) heat-inactivated fetal bovine serum (FBS) (Life Technologies, Grand Island, NY, USA) and antibiotics (100 U/mL penicillin and 100 U/mL streptomycin). Cells were maintained at 37 °C in a 5% CO_2_ incubator. U937 cells were incubated overnight with 100 ng/mL PMA for differentiation prior to LPS stimulation.

### 2.3. Protein Preparation and Binding Analysis of NLRP3 and LMT2368

The NLRP3 fragment (aa 134–676) was cloned into pGEX4T-1 and expressed in BL21-codon plus (DE3) *E. coli.* GST-NLRP3 was purified using GSH agarose. We determined the binding affinity of LMT2368 for NLRP3 using the Octet R8 system, with GST-NLRP3 captured on biosensors. The association and dissociation of LMT2368 (2.5–40 μM) were monitored, and the *K*_D_ value was calculated from kinetic constants.

### 2.4. NLRP3 Inflammasome Stimulation

J774A.1 and PMA-treated U937 cells were primed with 1 μg/mL LPS for 6 h. The cells were incubated with LMT2368 for 1 h and subsequently stimulated with 5 mM ATP or 5 μM nigericin for 30 min, respectively. The supernatants and cell lysates were collected for subsequent analysis.

### 2.5. Cell Viability and Cytotoxicity Assay

J774A.1, U937, and peritoneal macrophages were cultured in 96-well plates. The cells were treated with various concentrations of LMT2368 for 24 h. Briefly, the cells were washed with PBS, and 100 μL of CCK8 solution was added to each well. After 2 h of incubation at 37 °C, the absorbance at 490 nm was measured with a microplate reader. The data were analyzed according to the manufacturer’s instructions. The cell culture supernatants from the same experimental conditions were collected and analyzed by an LDH assay kit according to the manufacturer’s instructions. To determine pyroptotic cell death, the culture supernatants from inflammasome stimulation were collected and subjected to an LDH assay.

### 2.6. ASC Specks Formation

Peritoneal macrophages were cultured overnight on coverslips in 24-well plates. The cells were stimulated with LPS (1 μg/mL)/ATP (5 mM) in the presence of LMT2368. Then, the cells were washed with PBS and fixed with 4% paraformaldehyde (PFA). Next, the cells were blocked with PBS containing 10% FBS and 0.3% Triton-X 100, then incubated with anti-ASC antibodies overnight at 4 °C. The cells were washed with PBS and incubated with Alexa Flour 488-conjugated anti-mouse IgG antibodies for 1 h at room temperature. For nuclear staining, 4′,6-diamidino-2-phenylindole (DAPI) was applied to the coverslips for 20 min. After washing with PBS, the coverslips were placed on a glass slide. Fluorescent images were obtained using a Leica TCS SP5 laser scanning microscope (Wetzlar, Germany).

### 2.7. ASC Oligomer Assay

Peritoneal macrophages were seeded in 35 mm dishes overnight and stimulated with the inflammasome activators described above. Then, the cell extracts were harvested with lysis buffer (20 mM HEPES, pH 7.5, 150 mM KCl, 1% NP-40, and protease inhibitors) and centrifuged at 15,000 rpm for 15 min at 4 °C. After removing the supernatants, the pellets were washed with PBS and resuspended in 500 μL of PBS. Next, 2 mM disuccinimidyl suberate (DSS) was added, and the samples were incubated for 30 min at room temperature. The cross-linked samples were then centrifuged at 15,000 rpm for 15 min at 4 °C. The supernatants were removed, and the pellets were resuspended with 1.5× protein loading buffer. The samples were boiled at 100 °C for 10 min and subjected to SDS-PAGE and Western blotting with anti-ASC antibodies.

### 2.8. Co-Immunoprecipitation Assay

In order to investigate protein complex formation, co-immunoprecipitation (CO-IP) was conducted with cells expressing the proteins. HEK293 cells were transfected with FLAG-tagged NLRP3 and EGFP-tagged ASC1, which were obtained from Sinobiological (Beijing, China), as well as an empty vector for the control group. Twenty-four hours after transfection, the cells were washed with cold PBS, and a lysis buffer containing a protease inhibitor cocktail was added. To assess the effect of LM2368 on the NLRP3 complex, cells expressing FLAG-tagged NLRP3 and HA-tagged NLRP3 were incubated with 10 μM LMT2368 for 1 h and then lysed with the lysis buffer. The lysates were centrifuged, and the supernatants were incubated with anti-FLAG antibody-conjugated beads for 2 h at 4 °C. The beads were washed with the lysis buffer, resuspended in SDS sample buffer, and boiled. The samples were analyzed by Western blotting with ASC or NLRP3 antibodies.

### 2.9. Propidium Iodide (PI) Staining

J774A.1, or peritoneal macrophages, were cultured in 96-well plates overnight. The cells were stimulated with LPS (1 μg/mL)/ATP (5 mM) in the presence of LMT2368. Then, the cells were stained with PI for 15 min and DAPI for 20 min at room temperature. Fluorescent images were obtained using the EVOS M5000 microscope imaging system (Thermo Fisher Scientific, Waltham, MA, USA).

### 2.10. RT-PCR and Quantitative RT-PCR

Total RNA was extracted from lung tissues or cells using TRIzol (Invitrogen, Waltham, MA, USA). For lung tissues, each sample was homogenized in 1 mL TRIzol with a tissue grinder (Axygen, Union City, CA, USA), whereas for cells, the samples in TRIzol were pipetted for 2 min. RNA concentration was determined using a NanoDrop One C (Thermo Fisher Scientific, Waltham, MA, USA). Subsequently, 3 μg of total RNA was subjected to cDNA synthesis using reverse transcriptase (Promega, Madison, WI, USA).

For RT-PCR, the PCR mixture contained cDNA, Taq DNA polymerase, buffer, dNTPs, and primer pairs. Sequences of primers are shown as follows: TNF-α (5′-ACA AGC CTG TAG CCC ACG-3′ and 5′-TCC AAA GTA GAC CTG CCC-3′), IL-6 (5′-CAA GAA AGA CAA AGC CAG AGT CCT T-3′ and 5′-TGG ATG GTC TTG GTC CTT AGC C-3′), IL-1β (5′-TGC AGA GTT CCC CAA CTG GTA CAT C-3′ and 5′-GTG CTG CCT AAT GTC CCC TTG AAT C-3′), NLRP3 (5′-AGG AGT GGC TAA GGA CCA AGA-3′ and 5′-G ATA ACG CAC TAG GTT TGC CGA-3′), and GAPDH (5′-GAT GGG TGT GAA CCA CGA GAA-3′ and 5′-GAG CCC TTC CAC AAT GCC AA-3′). The PCR reaction was performed on a thermal cycler (SimpliAmp Thermal Cycler, Thermo Fisher Scientific, Waltham, MA, USA) using the following conditions: 95 °C, 5 min; 30 cycles (95 °C, 30 s; 58 °C, 30 s; 72 °C, 50 s); 72 °C, 10 min. PCR products were separated on a 1.5% agarose gel by electrophoresis and imaged. For QRT-PCR, SYBR Green mixture (iQTM SYBR^®^ Green Supermix, Bio-Rad, Hercules, CA, USA) and an iCycler PCR thermocycler (Bio-Rad, Hercules, CA, USA) were used along with target gene-specific primer sets designed with Primer-BLAST (NCBI, Bethesda, Rockville Pike, USA). Sequences of primers are shown as follows: TNF-α (5′-CCG ACT ATC TCG ACT TTG CC-3′ and 5′-GAT GTT CGT CCT CCT CAC AG-3′), IL-6 (5′-GGC TGC AGG ACA TGA CAA CT-3′ and 5′-ATC TGA GGT GCC CAT GCT AC-3′), IL-1β (5′-CTT CGA GGC ACA AGG CAC AA-3′ and 5′-TTC ACT GGC GAG CTC AGG TA-3′), NLRP3 (5′-CTC TGC GTC AAC CCA GAA GT-3′ and 5′-TTA GCC ATC TTG AAC AAT TTC-3′), and GAPDH (5′-CTCTGCTCCTCCTGTTCGAC-3′ and 5′-AATCCGTTGACTCCGACCTT-3′). The mRNA amounts of each gene were normalized to the mRNA level of glyceraldehyde 3-phosphate dehydrogenase (GAPDH).

### 2.11. LPS-Induced Acute Lung Injury Model

Eight-week-old female C57BL/6 mice weighing between 20 and 22 g were procured from KoaTech (Gyeonggi-Do, Korea). We maintained stringent adherence to ethical standards throughout all animal experiments and procedures, in full compliance with the Institutional Animal Care and Use Committee (IACUC) protocol at Korea University (KOREA-2024-0135). We ensured strict alignment with pertinent guidelines and regulations governing animal research. The entire cohort of mice was randomly divided into five different groups of 12 mice each: (1) a vehicle group that received 0.9% saline; (2) an LPS group treated with 1 mpk LPS; (3) an LPS + 5 mpk LMT2368 group treated with a combination of 1 mpk LPS and 5 mpk LMT2368; (4) an LPS + 20 mpk LMT2368 group treated with a combination of 1 mpk LPS and 20 mpk LMT2368; and (5) an LPS + 50 mpk LMT2368 group treated with a combination of 1 mpk LPS and 50 mpk LMT2368.

For the experiments, the mice received intraperitoneal injections of LMT2368 at concentrations of 5, 20, and 50 mpk, respectively, 30 min before the intratracheal instillation of 1 mpk LPS. The vehicle and LPS groups received saline injections. Twelve hours after LPS administration, an additional i.p. injection of LMT2368 was administered. Twenty-four hours after LPS administration, bronchoalveolar lavage fluid (BALF) and lung tissue were collected for reverse transcription-polymerase chain reaction (RT-PCR) analysis, Western blot analysis, determination of the wet/dry ratio, evaluation of immune cell infiltration and cytokine secretion, histological examination, and immunofluorescence analysis.

### 2.12. Bronchial Alveolar Lavage Fluid Analysis

To obtain bronchoalveolar lavage fluid (BALF) from each group, a tracheal cannula was used to flush the airways twice with 1 mL of a saline solution. The collected BALF was centrifuged at 2000× *g* for 15 min at 4 °C. The resulting supernatant was used to assess total protein and cytokine levels. Meanwhile, the pellet obtained after centrifugation was carefully spread onto slides for cell counting and classification of the BALF samples.

### 2.13. ELISA

J774A.1 cells were seeded in 96-well plates. The cells were pretreated with LMT2368 for 1 h and incubated with 1 μg/mL LPS for 6 h. Then, the culture supernatant was collected, centrifuged, and applied to an ELISA for TNF-α and IL-6 levels. For IL-1β, cells were primed with 1 μg/mL LPS for 6 h, then pretreated with different concentrations of LMT2368 for 1 h, and finally stimulated with 5 mM ATP or 5 μM nigericin for 30 min. The cell culture supernatant was collected, and the IL-1β levels were detected using an ELISA kit (Invitrogen, Waltham, MA, USA) according to the manufacturer’s instructions. The levels of TNF-α, IL-6, and IL-1β in BALF were also measured using the same ELISA kits.

### 2.14. Immunohistochemistry

After washing to remove the Optimal Cutting Temperature (OCT) compound from cryopreserved tissues by washing, 5 μm thick sections underwent a blocking step using 10% fetal bovine serum (FBS) at room temperature for 30 min. Then, the sections were exposed to specific primary antibodies either at room temperature for 3 h or at 4 °C overnight. After incubation, the sections underwent a triple wash using PBS containing 0.1% Tx-100 (PBST), with each wash lasting 10 min. Then, the sections were incubated with Alexa Flour 488-conjugated antibodies in a humid, dark chamber for 1 h. Subsequent washes with PBST for 10 min each were conducted. Next, the sections were incubated with anti-CD31 antibodies, an endothelial marker, for 1 h and washed with PBST. For nuclear staining, DAPI was applied to sections for 20 min. These sections were then covered with glass coverslips. Fluorescent imaging was carried out employing a Leica TCS SP5 laser scanning microscope (Wetzlar, Germany).

### 2.15. Histological Analysis

A total of 24 h after LPS treatment, mice were humanely euthanized, and their lung tissues were infused with a solution containing 4% paraformaldehyde (PFA) before being carefully preserved in this fixative solution. Subsequently, these tissues underwent embedding in paraffin. They were then sectioned into slices with a thickness of 5 μm. Hematoxylin and eosin (H&E) staining was then performed on lung sections. Brightfield images were obtained using a ZEISS Axio Scan.Z1 system (Carl Zeiss, Jena, Germany). Subsequent analysis of the captured images was conducted to detect and evaluate histopathological alterations present within the lung tissues.

The assessment of lung injury scores was conducted for various parameters, including the presence of neutrophils in either interstitial or alveolar spaces, thickening of alveolar septa, alveolar venous congestion, and accumulation of proteinaceous debris within airspaces. The severity of each parameter was recorded and categorized into one of three grades: normal (score of 0), mild-to-moderate (score of 1), or severe (score of 2). The summation of the individual scores from the five distinct parameters was then used to derive a comprehensive final score, which served to quantify the extent of lung injury.

### 2.16. Preparation of Peritoneal Macrophages

C57BL/6 mice were injected intraperitoneally with 1.5 mL of a sterile 3% thioglycollate solution. Four days later, the mice were sacrificed, and the peritoneal macrophages were collected with cold PBS. After centrifugation, the cells were cultured in complete RPMI-1640 medium at a density of 1 × 10^6^ cells/mL and incubated for 1 h at 37 °C. The non-adherent cells were then washed off with PBS, and the adherent macrophages were used for the experiments.

### 2.17. Western Blotting

Total proteins were extracted from lung tissues or cells using a lysis buffer (50 mM Tris-HCl, pH7.5, 150 mM NaCl, 10 mM NaF) with a protease inhibitor cocktail. The extracts were subjected to centrifugation at 15,000 rpm for 15 min at 4 °C. Protein concentrations were quantified employing a Bradford protein assay kit from Bio-Rad. To proceed, cell lysates underwent denaturation through the addition of sodium dodecyl sulfate (SDS) sample buffer, followed by boiling at 100 °C for 10 min. Proteins (10 µg for each sample) were then separated using 10% SDS-PAGE gels. After electrophoresis, proteins were transferred onto nitrocellulose membranes. These membranes were subsequently probed with appropriate primary and secondary antibodies. The resulting signals were detected using an enhanced chemiluminescence (ECL) assay kit (GE Healthcare, Chicago, IL, USA). The imaging process was conducted using an e-BLOT Touch Imager (e-BLOT, Shanghai, China).

### 2.18. Molecular Docking

The crystal structure of the NLRP3 NACHT domain was obtained from the Protein Data Bank (PDB ID: 7ALV) and prepared using the Protein Preparation Wizard in Maestro Schrödinger 2023.4. The crystal structure was prepared under default conditions in a step that included adding missing hydrogen atoms, assigning bond orders, and removing water molecules beyond 5 Å from the binding site. Next, MCC950 and LMT2368 under default conditions using Maestro’s LigPrep module. Molecular docking was performed using the induced fit protocol in the Schrödinger Suite in order to take into account the inherent flexibility of NLRP3. Visualization of the binding states was carried out using the Discovery Studio Visualizer program.

### 2.19. Statistical Analysis

Statistical analyses were conducted using PRISM9 software (Prism 9.0.0, GraphPad, La Jolla, CA, USA). The mean values are presented as means ± standard deviation (SD). The significance of the data was determined with Student’s *t*-test and/or one-way or two-way analysis of variance (ANOVA), followed by the Bonferroni post hoc test as applicable. Statistical significance was considered when the *p*-value was less than 0.05. All experiments were performed in triplicate, unless explicitly specified otherwise.

## 3. Results

### 3.1. LMT2368 Potently Decreases IL-1β Release by NLRP3 Binding

**Figure 1:**
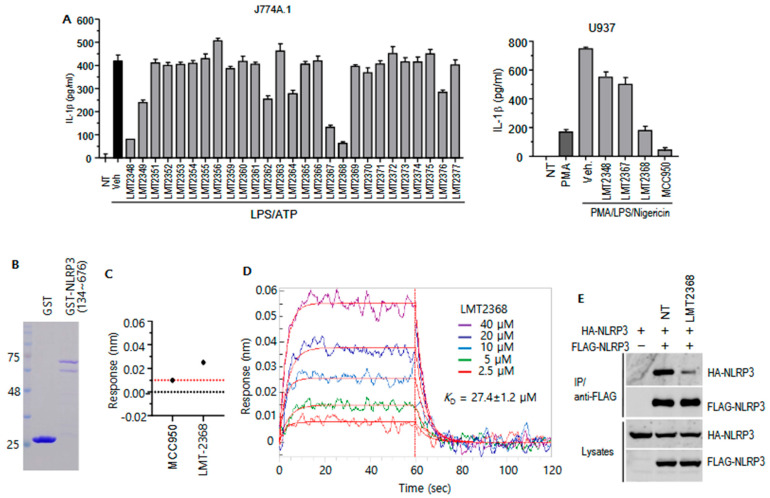
Screening the NLRP3 inhibitors with a cell-based assay and analysis of direct binding of LMT2368 and NLRP3. (**A**). ELISA for IL-1β secreted by inflammatory stimulation. LPS-primed J774A.1 cells were pretreated with the compounds and then stimulated with ATP. Culture supernatants were collected, centrifuged, and applied to ELISA for IL-1β. The inhibition efficiency of selected compounds was confirmed in U937 cells treated with PMA/LPS/Nigericin. NT: non-treatment, Veh.: DMSO-treated *p* < 0.01 vs. vehicle treatment (**B**). Purification of GST-fusion proteins. The pGEX4T-1 vector containing the gene fragment covering the NATCH domain of NLRP3 was introduced to BL21-codon plus (DE3) *E. coli*. After IPTG induction, cells were harvested, and the proteins were extracted with lysis buffer. The proteins purified with GSH agarose were applied to SDS-PAGE and Coomassie brilliant blue staining. (**C**). Direct binding of NLRP3 and LMT2368. GST-NLRP3 (aa 134~676) or GST was captured with Octet^®^ GST Biosensors (Satorius, Goettingen, Germany) and the binding kinetics were measured through association and dissociation of 10 μM LMT2368 or MCC950 in assay buffer. (**D**). Dose-dependent interaction. The captured proteins were incubated with different concentrations of LMT2368. The binding graph was obtained using a double reference subtraction protocol and analysis with Octet^®^ BLI analysis software (V13.0) provided by the company. (**E**). LMT2368 inhibits dimerization of NLRP3. Cells expressing FLAG-NLRP3 and HA-NLRP3 were incubated with LMT2368 for 1 h. The lysates were subjected to co-immunoprecipitation with anti-FLAG agarose and subsequent Western blotting. Small bars on the left side of the blots indicate molecular weights of 135 kDa and 100 kDa.

Inflammasome activation in macrophages leads to the cleavage of pro-IL-1β and the subsequent release of mature IL-1β. In light of the constraints imposed by MCC950, a library of urea analogs was screened for NLRP3 inhibition (Supplementary Table S1). To examine the inhibitory effects of the urea analogs on inflammasome activation, J774A.1 and U937 cells were adopted as the cellular inflammation model system. These cells are monocytic cell lines from mouse and human, respectively. ATP is a well-known NLRP3 inflammasome activator in LPS-primed macrophages, resulting in the subsequent activation of caspase-1 and IL-1β secretion. J774A.1 cells secreted IL-1β in response to LPS pretreatment and ATP stimulation. However, U937 cells were not suitable for studying the NLRP3 inflammasome in this condition. Instead, nigericin, a potassium ionophore derived from the Gram-positive bacterium *Streptomyces hygroscopicus,* induced IL-1β secretion when the U937 cells were differentiated with phorbol 12-myristate 13-acetate (PMA) and subsequently primed with LPS. In J774A.1 cells, IL-1β secretion was potently decreased by LMT2348, LMT2367, and LMT2368 (Figure 1A, left graph). Among these, only LMT2368 demonstrated robust inhibitory effects in both mouse and human cell lines, indicating the potential for cross-species efficacy. (Figure 1A). In order to investigate direct interaction between the compound and NLRP3, the NLRP3 protein fragment containing the NATCH domain (His220~Phe650) was expressed as a GST-fusion form in E. coli and purified with GSH agarose (Figure 1B). The interaction was determined through biolayer interferometry (BLI) assay as described in the experimental procedures. A notable observation is the higher binding efficiency of LMT2368 compared to MCC950 at a concentration of 10 μM, as depicted in Figure 1C. As the concentration of LMT2368 increased, the binding reaction also increased with *K*_D_ = 27.4 ± 1.2 μM (Figure 1D), indicating that LMT2368 binds to NLRP3 with specificity, although the BLI assay with GST-tagged NLRP3 fragment is less sensitive to show low affinity. Co-immunoprecipitation with cells expressing HA- and FLAG-tagged NLRP3 showed that dimerization of NLRP3 was significantly inhibited in the presence of LMT2368, suggesting that the compound blocked NLRP3 inflammasome formation (Figure 1E)

### 3.2. Mechanistic Insights into NLRP3 Inhibition by MCC950 and LMT2368

**Figure 2:**
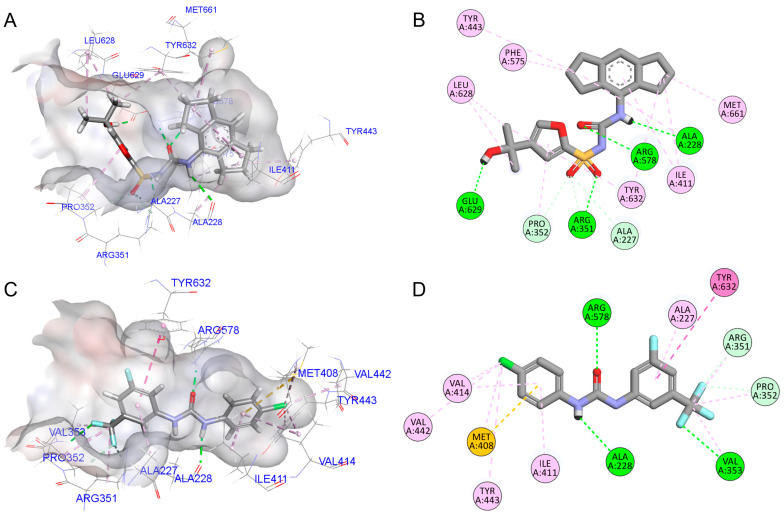
Binding models of MCC950 and LMT2368 in the binding pocket of NLRP3. (**A**) Binding model (docking study) between MCC950 (orange) and NLRP3 (PDB: 7ALV). Green dashed lines indicate H-bonds. (**B**) Protein-ligand interactions of MCC950 with NLRP3. Green-dashed lines are H-bonds, pink-dashed lines are hydrophobic interactions, and magenta-dashed lines are Pi-Pi interactions. (**C**) Binding model (docking study) between LMT2368 (magenta) and NLRP3 (PDB: 7ALV). (**D**) Protein-ligand interactions of LMT2368 with NLRP3.

The crystal structure of the NLRP3 NACHT domain in complex with a potent inhibitor was obtained from the protein data bank (PDB: 7ALV). The crystal structure revealed multiple molecular interactions predicted to be responsible for the exhibited biological activity. The docking of MCC950 with NLRP3 revealed the establishment of three hydrogen bonds between MCC950 and amino acid residues of the NLRP3 protein. The first hydrogen bond was established between the carbonyl oxygen of Ala228 and the NH group of the urea moiety. The second hydrogen bond was formed between the guanidine side chain of Arg578 and the urea’s carbonyl oxygen. The third hydrogen bond was formed between the side chain carbonyl of Glu629 and the hydroxyl group of the inhibitor. Furthermore, the hexahydroindacene moiety of MCC950 occupied a hydrophobic pocket within the NLRP3 binding site and interacted with Ile411, Leu413, and Met661 (Figure 2A,B). This interaction served to further stabilize the MCC950 binding with NLRP3. The binding affinity of MCC950 was predicted to be −6.961 kcal/mol.

Induced-fit docking (IFD) of LMT2368 yielded 14 poses, of which two poses exhibited promising binding configurations upon visual inspection, thus warranting further evaluation. The top-scoring pose for LMT2368 revealed that LMT2368 occupied the same pocket as MCC950 with an enhanced docking score of –7.950 kcal/mol. This increase in the binding affinity was predicted to be due to several key interactions formed between LMT2368 and NLRP3 (Figure 2C,D). In a similar fashion to MCC950, LMT2368 maintains the hydrogen bonds with Arg578 and Ala228 amino acid residues of the NLRP3 binding site. Concurrently, LMT2368 established an additional hydrogen bond with the amino group of Val353 via the trifluoromethyl (CF_3_) moiety. The result obtained in this study is at odds with the findings of MCC950, which exclusively engages in hydrophobic contacts at this specific site. Furthermore, the meta-trifluorobenzyl urea core of LMT2368 was predicted to closely mimic the binding orientation of MCC950′s sulfonylurea group. Conversely, the replacement of the hexahydroindacene moiety of MCC950 with a 4-chlorobenzene moiety in LMT2368 led to the retention of the same hydrophobic pocket, with the result that it interacted with Ile411, Val414, Val442, and Tyr443.

The second promising IFD result occupies the same binding site as MCC950 but displays a different interaction profile, resulting in a slightly lower binding affinity (−6.721 kcal/mol), comparable to MCC950. This reduced affinity is partly due to an unfavorable electrostatic interaction, wherein an additional NH group in the urea scaffold forms a repulsive contact with Ala228′s amino group. However, LMT2368 maintained hydrophobic interactions with Ile411, Val414, Val442, and Tyr443. In contrast to LMT2368, it does not form a hydrogen bond with Val353, instead relying exclusively on hydrophobic contacts. These structural analyses underscore the potential for enhancing the potency of NLRP3 inhibitors through the judicious implementation of chemical modifications. The introduction of trifluoromethyl groups has been demonstrated to facilitate additional hydrogen bonding while preserving essential interactions and the core binding mode. These insights provide valuable guidance for the design of next-generation NLRP3 inhibitors with improved affinity and selectivity.

### 3.3. LMT2368 Attenuates Inflammasome Activation in Monocytic Cell Lines

**Figure 3:**
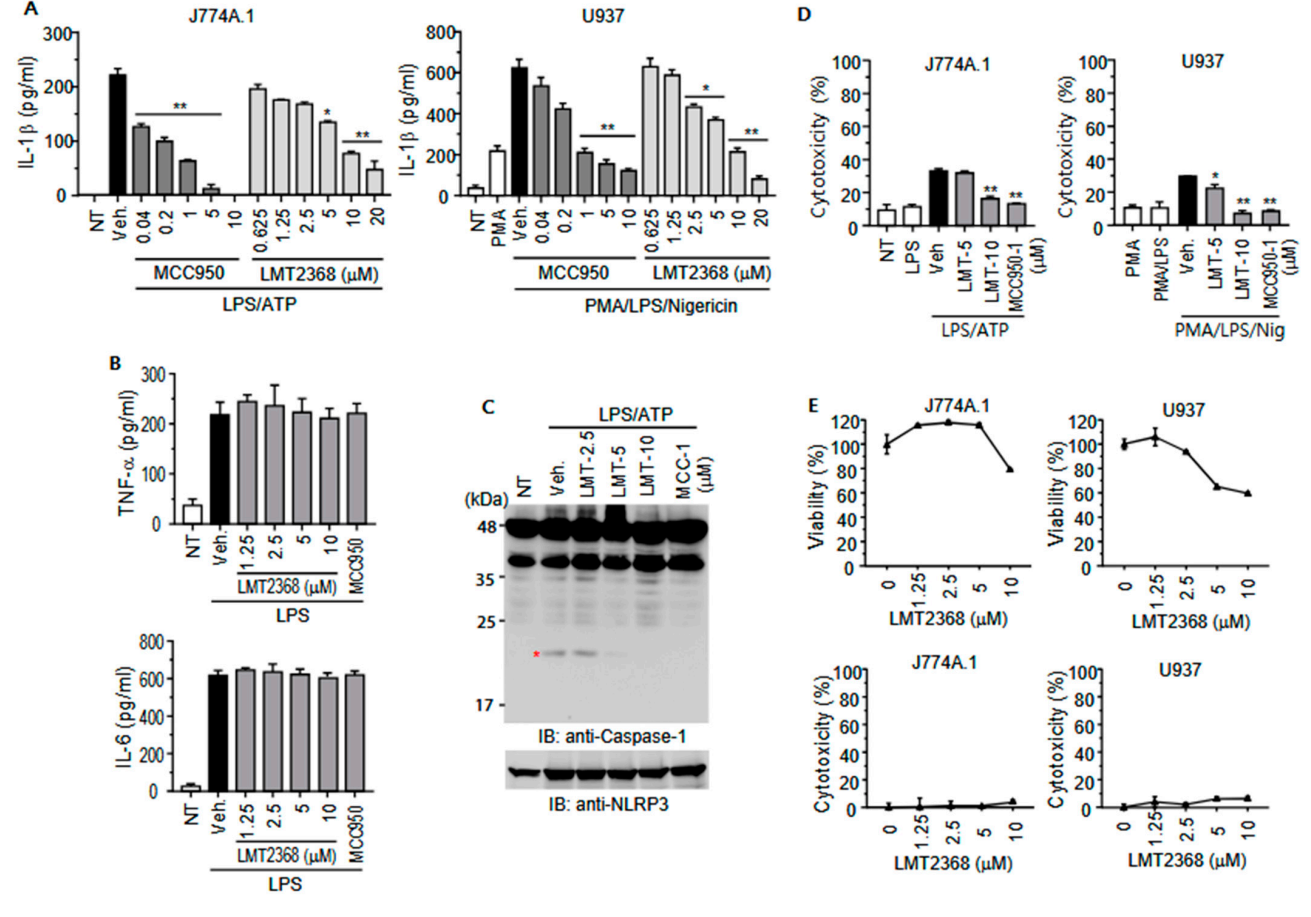
LMT2368 downregulates inflammasome activation in monocytic cell lines. (**A**) Dose dependency of LMT2368 inhibition. J774A.1 and PMA-treated U937 cells were stimulated with LPS for 6 h and incubated with different concentrations of MCC950 or LMT2368 1 h before ATP or Nigericin treatment. After 30 min, culture supernatants were collected and applied to ELISA for IL-1β. * *p* < 0.05; ** *p* < 0.01 vs. vehicle treatment. (**B**) LMT2368 has no effect on LPS-stimulated secretion of TNF-α and IL-6. J774A.1 cells were treated with LPS in the presence of different concentrations of LMT2368, and the culture supernatants were collected and applied to ELISA for TNF-α and IL-6. (**C**) LPS/ATP-stimulated caspase-1 cleavage is suppressed by LTM2368. An asterisk indicates cleaved caspase-1. (**D**) Culture supernatants of J774A.1 and U937 cells were collected 30 min after final treatment with inflammasome stimulators and applied to the LDH assay. (**E**) Both cells were treated with different concentrations of LMT2368 for 24 h and subjected to CCK-8 assay. The culture supernatants were collected and applied to the LDH assay.

Subsequent examination of the inhibitory effect of LMT2368 on IL-1β secretion revealed a dose-dependent decrease in secreted IL-1β in both mouse and human monocytic cell lines, albeit with slightly different efficiencies. In contrast to the results of the direct binding assay, the inhibitory activity of LMT2368 was found to be lower than that of MCC950. Furthermore, MCC950 demonstrated a more pronounced inhibitory effect on cytokine secretion in J774A.1 cells compared to U937 cells. However, the inhibitory activity of LMT2368 was more pronounced in U937 cells. This result suggests that the binding properties of LMT2368 and MCC950 to NLRP3 were different from each other (Figure 3A).

In the priming step of activating the NLRP3 inflammasome, LPS has been shown to induce the expression of pro-inflammatory cytokines such as TNFα and IL-6 as well as inflammasome components including NLRP3 and IL-1β [28]. To determine the target specificity of LMT2368, J774A.1 cells and mouse peritoneal macrophages were treated with LPS in the presence of LMT2368. RT-PCR using gene-specific primers revealed that the expression of all tested genes was induced by LPS, regardless of LMT2368 or MCC950 treatment (Supplementary Figure S1). Moreover, LMT2368 exhibited no effect on the secretion of TNF-α and IL-6 from J774A.1 cells in response to the stimuli (Figure 3B). These results further validate that LMT2368 inhibits inflammasome activation by directly targeting NLRP3.

It has been established that the cleavage of pro-caspase-1 by the NLRP3 inflammasome complex is a prerequisite for the cleavage and release of pro-IL-1β. To this end, cell extracts from treatment with stimulators and various concentrations of the compounds were applied to Western blotting with anti-casapse-1 antibodies. The cleaved caspase-1 appeared at around the 20 kDa position by LPS and ATP treatment; however, the band disappeared in the presence of 5 and 10 μM LMT2368 as well as MCC950. In the stimulation conditions, the expression of NLRP3 increased, yet this increase was not affected by the compounds (Figure 3C).

The inflammasome is activated by PAMPs and the opening of channels, which in turn induce the death of affected cells, a process referred to as pyroptosis [29]. The ion influx by ATP or nigericin in cells primed with LPS led to approximately 30% cell death in both cell lines, which was decreased by LMT2368 and MCC950 (Figure 3D). Next, to ascertain the toxicity of LMT2368 itself, J774A.1 and U937 cells were exposed to varying concentrations of the compound. The CCK-8 assay was used to determine the viability of the cells. The results showed a decrease in viability at 10 μM concentration in J774A.1 cells and a more pronounced decrease in U937 cells. However, the LMT2368-dependent LDH release to the media was barely detected in both cells, suggesting that this compound is relatively safe, while it somehow affects cell growth (Figure 3E).

### 3.4. LMT2368 Inhibits Inflammasome Activation in Primary Macrophages

**Figure 4:**
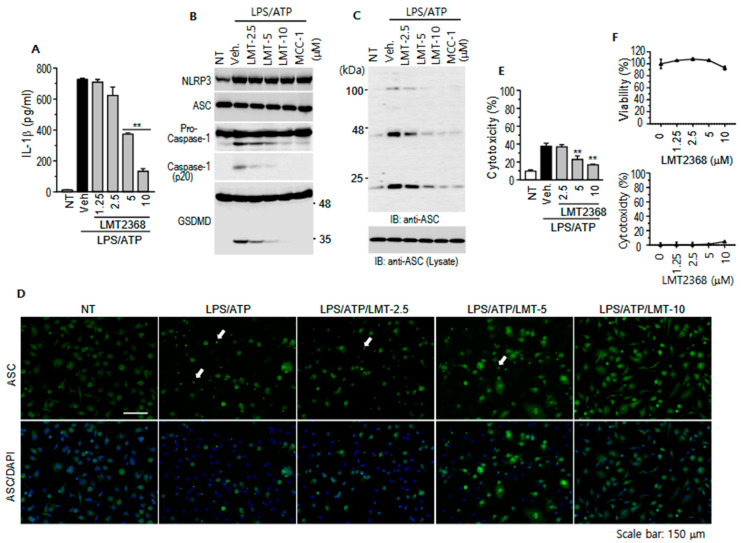
LPS/ATP-stimulated inflammasome activation in peritoneal macrophages was suppressed by LMT2368. (**A**) Peritoneal macrophages derived from thioglycolate were seeded in a 96-well plate. After 24 h, attached cells were treated with LPS for 6 h and subsequently stimulated with ATP for 30 min in the presence of different concentrations of LMT2368, and then culture supernatants were collected and subjected to ELISA for IL-1β. ** *p* < 0.01 vs. vehicle treatment. (**B**) Cells in 35 mm dishes were treated with LPS/ATP in the presence of LMT2368 and harvested with lysis buffer and subjected to immunoblotting with appropriate antibodies. (**C**) ASC- oligomerization was inhibited by LMT2368. After inflammasome stimulation, cells were lysed and centrifuged to remove soluble proteins. PBS-washed pellets were resuspended with PBS and incubated with DSS, a crosslinker. After centrifugation, pellets were solubilized with 1.5× protein loading buffer and subjected to SDS-PAGE and Western blotting with anti-ASC antibodies. Lower is the blot for total ACS proteins in each sample. (**D**) ASC specks were decreased by LMT2368. Peritoneal macrophages on coverslips were treated with LPS/ATP in the presence of LMT2368, fixed with 4% PFA, and incubated with anti-ASC antibodies. Fluorescence signals were visualized with Alexa Flour 488-conjugated anti-mouse IgG antibodies and DAPI. White arrows indicate the ASC specks. (**E**) LPS/ATP-stimulated pyroptosis was inhibited by LMT2368. Thirty minutes after ATP treatment, the culture supernatants were collected and applied to the LDH assay. ** *p* < 0.01 vs. vehicle treatment. (**F**) Cells were treated with different concentrations of LMT2368 for 24 h and subjected to CCK-8 assay. The culture supernatants were collected and applied to the LDH assay.

The established monocytic cell lines have been observed to undergo a process of genetic drift, resulting in the loss of their original characteristics as immune cell lineages. This phenomenon is attributed to the fact that these cell lines have already undergone transformation and immortalization. Consequently, the screening of compounds to regulate specific signaling pathways and cellular responses may be a viable approach. However, it should be noted that observed responses may not occur in the same manner as they did in the original cells. In order to circumvent the limitations associated with experiments utilizing cell lines, we isolated peritoneal macrophages, which were migrated by thioglycolate, and employed them to confirm the inhibitory activity of LMT2368 on NLRP3. LPS- and ATP-stimulated IL-1β secretion in these primary macrophages was found to be significantly higher compared to that observed in J774A.1 cells. The inhibitory effect of LMT2368 was successfully reproduced, and the efficiency was found to be even more pronounced (Figure 4A). Subsequent analysis via Western blots, employing specific antibodies, revealed that LPS priming induced NLRP3 expression but not ASC, pro-caspase-1, and GSDMD, which is similar to the results in previous reports. Subsequent ATP stimulation induced cleavage of pro-caspase-1 and GSDMD, indicating the activation of the NLRP3 inflammasome. However, the cleaved proteins decreased with increasing LMT2368, implying that LMT2368 inhibited inflammasome-dependent caspase-1 activation (Figure 4B). NLRP3-mediated ASC oligomerization is a critical event in NLRP3 inflammasome activation. ASC oligomers were determined by adding a cross-linker to cell extracts and subsequent precipitation by centrifugation. Western blots with anti-ASC antibodies showed that ASC monomers and higher-order complexes were significantly increased by stimulation with LPS and ATP, while the complex formation was attenuated by LMT2368 in a dose-dependent manner (Figure 4C). Furthermore, ASC oligomerization was observed by immunostaining with anti-ASC antibodies. Upon LPS and ATP stimulation, a significant increase in the number of condensed green specks was observed within peritoneal macrophages. However, the numbers of specks were decreased with increasing LMT2368 (Figure 4D), suggesting that LMT2368 blocks NLRP3-mediated ASC oligomerization and inflammasome activation. The targeting of the NATCH domain in NLRP3 by LMT2368 has the potential to inhibit inflammasome activation, irrespective of the interaction between NLRP3 and ASC through PYD in each protein. This was confirmed in a co-immunoprecipitation experiment (Supplementary Figure S2).

The pyroptotic death by inflammasome activation was also clearly observed in the peritoneal macrophages, which was relatively more efficiently inhibited by LMT2368 in comparison to the established cell lines (Figure 4E). In addition, the cytotoxic effect of LMT2368 itself appears to be relatively low according to the results from CCK-8 and LDH assays conducted on this primary cell. This finding is more pronounced than that observed in J774A.1 and U937 cells (Figure 3E and Figure 4F). The results from PI staining in J774A.1 cells and peritoneal macrophages further corroborated the finding that LMT2368 protects cells from pyroptotic death by inflammasome activation (Supplementary Figure S3).

### 3.5. LMT2368 Alleviates LPS-Induced Acute Lung Injury

**Figure 5:**
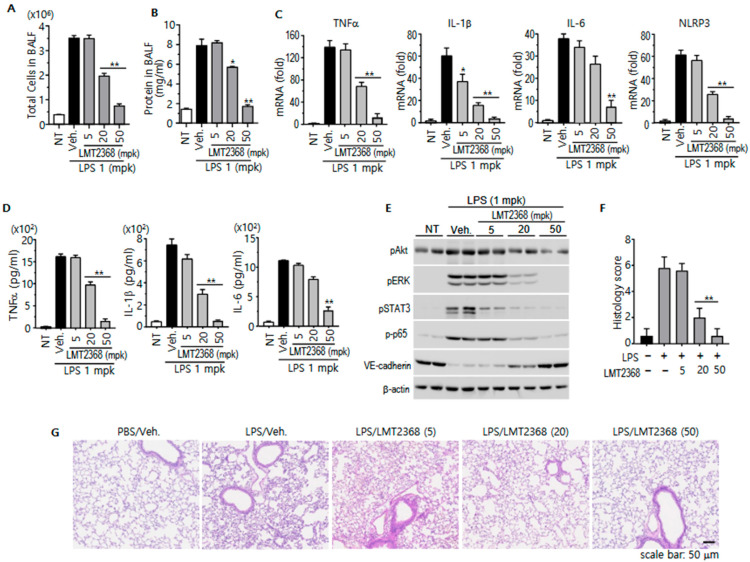
Anti-inflammatory effect of LMT2368 in LPS-stimulated ALI mice. (**A**). Total cells in BALF. PBS-washed lung fluids from each group were centrifuged. Pelleted cells were resuspended in PBS and counted under a light microscope. (**B**). Total protein amount in BALF. (**C**). Quantitative RT-PCR. Total RNAs isolated from lung tissues were subjected to reverse transcription and quantitative PCR with gene-specific primers. (**D**). ELISA for cytokines in BALF. (**E**). Proteins from lung tissues were extracted with lysis buffer and applied to Western blotting with appropriate antibodies. (**F**). Histological scores of lung tissues from each group. (**G**). Representative histological changes in the lung obtained from mice treated with LPS alone or LMT2368/LPS. H&E-stained tissues * *p* < 0.05; ** *p* < 0.01 vs. vehicle treatment.

LPS-induced lung injury is a widely utilized mouse model to study acute lung injury and acute respiratory distress syndrome, which is similar to the inflammatory responses observed in human patients [30,31]. In this model, NLRP3 inflammasome activation may be a critical step in the propagation of inflammatory distress throughout the body, as well as the lung. This assertion is supported by the established role of LPS as a prominent PAMP molecule [32]. Tracheal instillation of LPS causes the disruption of alveolar epithelial architecture, leading to fluid leakage and immune cell infiltration into the alveolar space. In the present study, 1 mg/kg LPS instillation augmented cell numbers infiltrated into the alveoli, as evidenced by the collection of these cells in bronchoalveolar lavage fluid (BALF). The stimulation also increased protein content in BLAF, indicating that the model system is well-established. Furthermore, LMT2368 led to a significant decrease in the number of infiltrated cells and the protein levels in BALF in a dose-dependent manner (Figure 5A,B).

Inflammatory responses are typically accompanied by increased levels of various inflammatory cytokines [33]. As previously reported, LPS instillation led to increases in early inflammatory cytokines such as TNFα, IL-1β, and IL-6 in lung tissues, which were clarified by quantitative RT-PCR and ELISA. LMT2368 treatment led to a significant decrease in these cytokines, albeit with slightly different efficiencies. However, it should be noted that the expression of these cytokines, as well as NLRP3, is stimulated by LPS/TLR4 signaling pathways. Nevertheless, these pathways are not directly affected by NLRP3 inflammasome activation (Figure 5C,D). These results may support the idea that NLRP3 inflammasome activation is a pivotal step in local and/or systemic inflammatory responses, with the potential to modulate the expression of pro-inflammatory cytokines.

The present study sought to explore the phosphorylation states of signaling molecules in response to inflammatory stimulation. To this end, lung tissue extracts were applied to Western blotting. LPS stimulation increased the phosphorylation of Akt, ERK, STAT3, and p65 of NF-kB, suggesting that not only TLR4 but also some cytokine receptors may be activated in the inflammatory condition [34,35,36]. The decrease in VE-cadherin levels may be indicative of a disruption in the integrity of the vascular endothelial layers in this condition. However, LTM-2368 treatment led to a decrease in the phosphorylation of the signaling molecules and a maintenance of VE-cadherin expression. This suggests that LMT2368 has anti-inflammatory activity and protects vascular structure to prevent body fluid leakage (Figure 5E).

The histological changes induced by LPS application were investigated in the lung tissue stained with hematoxylin and eosin (H&E) and scored based on the five parameters described in the experimental procedures. In comparison to the negative control group, the LPS-treated group exhibited an indication of substantial immune cell infiltration into the interstitial region, a partially thickened alveolar septum, and a reduction in alveolar space with collapse. The LPS-stimulated histological changes were relieved similarly to normal tissues in the groups treated with 20 and 50 mg/kg LMT2368 (Figure 5F,G).

**Figure 6:**
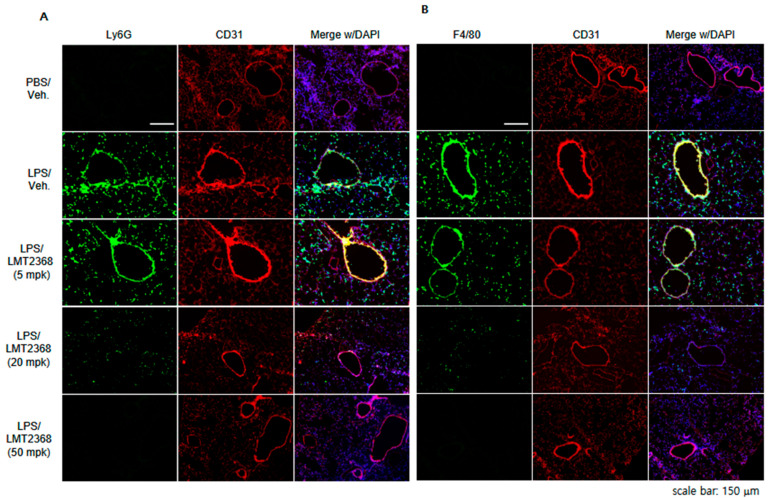
LMT2368 suppresses LPS-stimulated immune cell infiltration to lung tissues. Immunohistochemistry with antibodies for (**A**) neutrophil marker (Ly6G) or (**B**) macrophage marker (F4/80). Frozen sectioned lung slices from each group were stained with the specific antibodies.

According to immunohistochemical analysis with cell-type-specific antibodies, Ly6G- or F4/80-positive signals were significantly enriched in the lung treated with LPS, indicating that neutrophils and macrophages were infiltrated into the interstitial area by pathogenic stimulation. However, the immune cell accumulation was gradually decreased by increasing doses of LMT2368, possibly through the anti-inflammatory activity of the compound in the initial step of ALI (Figure 6A,B).

## 4. Discussion

Inflammation is a fundamental biological response for maintaining health and homeostasis by eliminating harmful stimuli, repairing damaged tissue, and initiating protective responses. However, dysregulated inflammation can be detrimental, contributing to the pathogenesis of many diseases through excessive cytokine and impaired healing processes. Therefore, the inflammatory response must be finely regulated to ensure optimal immune activation, preventing both inadequate and excessive reactions.

The NLRP3 inflammasome is a central component of the innate immune system, playing a pivotal role in initiating inflammatory responses. It has emerged as a promising therapeutic target for various inflammatory and autoimmune disorders, as its dysregulation is implicated in numerous inflammation-related diseases. Drug discovery efforts targeting NLRP3 have identified MCC950, a potent NLRP3 inhibitor; however, the drug development was halted in Phase II clinical trials due to hepatotoxicity, off-target effects, and pharmacokinetic limitations. Various sulfonylurea analogs of MCC950 have also been explored, but the autonomous function of the inflammasome poses challenges for long-term therapeutic applications targeting NLRP3 alone.

In this study, to overcome these limitations, we screened an in-house library of urea analogs, avoiding sulfonylurea scaffolds, and identified LMT2368 as a novel NLRP3 inflammasome inhibitor. Screening 27 compounds in J774A.1 cells revealed that LMT2348, LMT2367, and LMT2368 significantly decreased IL-1β secretion, with only LMT2368 showing robust inhibitory effects in human U937 cells, indicating cross-species activity. The specific inhibition by LMT2368 was confirmed using biochemical and cell-based assays, including by biolayer interferometry (BLI) assay, which demonstrated its binding to the NLRP3 NACHT domain. Although the binding affinity observed via BLI was modest compared to results from surface plasmon resonance (SPR) reported for MCC950, BLI offers a simpler qualitative method and still showed higher binding of LMT2368 compared to MCC950 [37,38].

Molecular docking studies revealed that LMT2368 and MCC950 share the same binding pocket within the NLRP3 NACHT domain. This finding suggests that despite structural modifications, the primary binding site remains conserved (Figure 2). Notably, LMT2368 exhibited a superior binding profile compared to the established NLRP3 inhibitor MCC950. This enhanced binding affinity suggests that structural optimization successfully maintained target specificity while improving molecular recognition and stability within the binding site. Structurally, LMT2368 lacks the (2-hydroxypropan-2-yl) furan moiety of MCC950, resulting in the loss of a key hydrogen bond with Glu629. Remarkably, LMT2368 compensates for this by forming a unique hydrogen bond between its trifluoromethyl (CF_3_) group and the backbone amino group of Val353—a relatively unusual interaction for CF_3_ groups, likely enabled by the electronegativity of fluorines. This alternative bonding may contribute to the superior binding stability suggested by docking. Moreover, the CF_3_ group is known to enhance pharmacokinetic properties such as lipophilicity and membrane permeability, potentially improving bioavailability compared to MCC950. Additionally, the removal of the furan moiety addresses a major metabolic liability seen with MCC950, where the furan ring undergoes cytochrome P450-mediated oxidative metabolism, causing unstable metabolites and reduced efficacy. The design of LMT2368 thus introduces improved metabolic stability alongside target specificity.

Activation of the NLRP3 inflammasome involves a priming step mediated mainly by NF-κB and MAP kinase signaling and a subsequent activation step culminating in caspase-1 cleavage, cytokine release, and pyroptotic cell death [39]. LMT2368 does not inhibit the priming phase—as corroborated by unchanged LPS-induced gene expression of inflammasome components and pro-inflammatory cytokines—but specifically inhibits inflammasome assembly and downstream caspase-1 activation, thereby suppressing IL-1β secretion. These findings underscore LMT2368’s targeted mechanism through direct interaction with NLRP3. Beyond the inflammasome, inflammatory signaling is tightly regulated by additional pathways that influence priming and activation. The proteasome system, for example, regulates key proteins, including IκB, facilitating NF-κB activation that primes inflammasome gene expression. Similarly, MAPK pathways (p38, JNK) contribute to NF-κB/AP-1 activation and thus promote transcription of NLRP3 and pro-IL-1β. Including these regulatory axes provides a broader understanding of inflammasome modulation and therapeutic targeting. Incorporation of recent insights regarding proteasome-mediated degradation of viral and bacterial proteins and MAPK’s role in sepsis and inflammation offers further context on the complexity of NLRP3 regulation and highlights potential combinational strategies for inflammatory disease management.

Inflammation underlies a broad spectrum of diseases, including cardiovascular and metabolic disorders and cancers, where it often acts as a secondary factor rather than the primary cause. Hence, while NLRP3 inhibitors may not serve as standalone cures for such conditions, they can alleviate symptoms by modulating immune cell-driven inflammation. This therapeutic potential is particularly pertinent for autoimmune diseases such as inflammatory bowel disease, atopic dermatitis, asthma, and acute inflammatory conditions like infection and sepsis.

To model acute inflammation and lung injury, we investigated LMT2368 in an LPS-induced acute lung injury (ALI) mouse model, which recapitulates key features of human ALI/ARDS, including immune cell infiltration, cytokine storm, and pulmonary edema [38,40,41,42,43]. LMT2368 significantly reduced immune cell infiltration and protein leakage to the alveolar space and pro-inflammatory cytokine levels (TNF-α, IL-1β, IL-6) in a dose-dependent manner. While LMT2368 did not modify priming-related gene expression in monocytic cells, it markedly attenuated phosphorylation of signaling molecules such as Akt, ERK, STAT3, and NF-κB p65 in lung tissues, suggesting modulation of downstream cytokine receptor signaling and preservation of vascular endothelial integrity (evidenced by VE-cadherin maintenance). Histological analyses further confirmed reduced infiltration of neutrophils and macrophages, underscoring LMT2368’s anti-inflammatory activity.

In summary, given the pivotal role of NLRP3 inflammasome activation in diverse inflammatory and autoimmune disorders, and considering LMT2368’s efficacy and improved pharmacological profile demonstrated in vitro and in vivo, this compound holds promise as a lead candidate for therapeutic development. Its application may extend to acute inflammatory syndromes featuring cytokine storms and respiratory distress, such as those seen in critical illnesses and infectious diseases, including COVID-19.

## Data Availability

The original contributions presented in this study are included in the article. Further inquiries can be directed to the corresponding author(s).

## Supplementary Materials

The following supporting information can be downloaded at https://www.mdpi.com/article/10.3390/pharmaceutics17101241/s1: Table S1: Synthesis of Urea derivatives LMT-2348-2377; Figure S1: LMT-2368 has no effect on LPS-stimulated gene expression; Figure S2: LMT2368 has no effect on the interaction of NLRP3 and ASC; Figure S3: LMT-2368 inhibits pyroptosis.

## Abbreviations

NLRP3: NOD-like receptor family pyrin domain containing 3; ALI, acute lung injury; PAMPs, pathogen-associated molecular patterns; DAMPs, damage-associated molecular patterns (DAMPs); ASC, apoptosis-associated speck-like protein containing a caspase-recruitment domain; LPS, lipopolysaccharide; BALF, bronchoalveolar lavage fluid; TNF-α, tumor necrosis factor-α; PFA, paraformaldehyde.
