# Supplementary material for: LMT2368 (1-(4-Chlorophenyl)-3-(3-fluoro-5-(trifluoromethyl)phenyl)urea) Negatively Regulates Inflammation by Inhibiting NLRP3 Inflammasome Activation
Source: Pharmaceutics. 2025 Sep 23;17(10):1241. doi: 10.3390/pharmaceutics17101241 (PMC12566734; doi:10.3390/pharmaceutics17101241)
Supplement: Supplementary file 1 [file pharmaceutics-17-01241-s001.zip › pharmaceutics-3860675-supplementary.pdf]

Supplementary materials

LMT2368 (1-(4-Chlorophenyl)-3-(3-Fluoro-5-(Trifluoromethyl)Phenyl)Urea) Negatively Regulates Inflammation by Inhibiting NLRP3 Inflammasome Activation

Thai Uy Nguyen [1], Su Jeong Kwon [2], Sunghoon Hurh [1], Ashok Kale [2], Jae Min Cho [2], Hossam Nada [2,3], Chang Seong Kim [2], Peela Induvadana [4], Beom Jin Park [5], Kyeong Lee [2,*], Yongseok Choi [4] and Jong-Ik Hwang [1,*]

Supplementary Table S1. Synthesis of Urea derivatives LMT-2348-2377

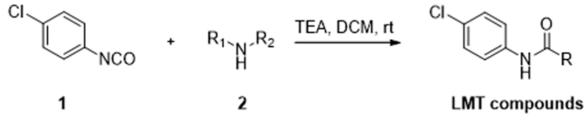

Compound	R	Compound	R	Compound	R
MCC950		LMT-2360	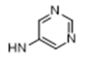	LMT-2370	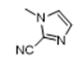
LMT-2348	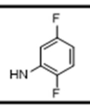	LMT-2361	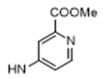	LMT-2371	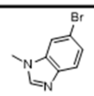
LMT-2349	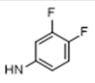	LMT-2362	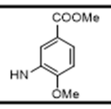	LMT-2372	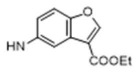
LMT-2351	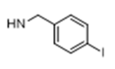	LMT-2363	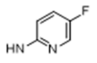	LMT-2373	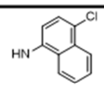
LMT-2352	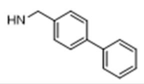	LMT-2364	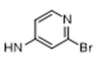	LMT-2374	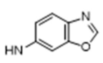
LMT-2353	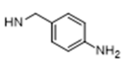	LMT-2365	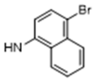	LMT-2375	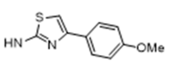
LMT-2354	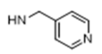	LMT-2366	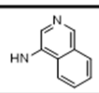	LMT-2376	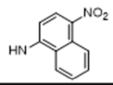
LMT-2355	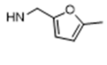	LMT-2367	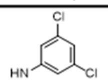	LMT-2377	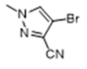
LMT-2356	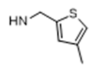	LMT-2368	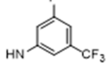		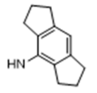
LMT-2359	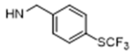	LMT-2369	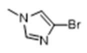		

General procedure

All commercial chemicals were reagent-grade and were used without further purification. Solvents were dried with standard procedures. All reactions were carried out under an atmosphere of dried argon in flame-dried glassware. The proton nuclear magnetic resonance (^{1}H NMR) spectra were determined on a Varian 500 MHz spectrometer (Varian Medical Systems, Inc., Palo Alto, CA, USA). The ^{1}H NMR data are presented in terms of peak multiplicities, where "s" represents a singlet, "d" indicates a doublet, "dd" for doublet of doublets, "t" indicates triplet, "q" represents quartet, "brs" for a broad singlet, and "m" indicates a multiplet. ^{13}C NMR spectra were recorded on a Varian 126 MHz spectrometer. Chemical shifts are expressed in δ values (ppm), while coupling constants (J) are reported in Hertz (Hz). The mass spectra were recorded using high-resolution mass spectrometry (HRMS) (electron ionization MS) on a JMS-700 mass spectrometer (Jeol, Japan) or by HRMS (electrospray ionization MS) on a G2 QTOF mass spectrometer. The products from all reactions were purified by flash column chromatography using silica gel 60 (230–400 mesh Kieselgel 60). Additionally, thin-layer chromatography on 0.25-mm silica plates (E. Merck; silica gel 60 F254) was used to monitor reactions.

General procedure for synthesis of Urea derivatives (LMT-2348- LMT-2377)

To a solution of 4-chlorophenyl isocyanate (1.0 equiv.) in Pyridine (5 mL) at room temperature, the amine (1.0 equiv.) was added. The mixture was stirred at room temperature for 1 h. After completion of reaction, the solvent was evaporated, and reaction mixture was neutralized using 2 M HCl solution. The solution was extracted using EtOAc (2*15 mL). The combined organic layers were washed with brine, dried over $MgSO_4$, and concentrated in vacuo. The residue was purified by silica gel column chromatography (n-hexane: EtOAc).

1-(4-chlorophenyl)-3-(2,5-difluorophenyl)urea (LMT-2348)

Isolated as a white solid (501 mg, 54% yield, Purification by column chromatography (20/1, petroleum ether/ethyl acetate); mp 225-227°C.; ^{1}H NMR (500 MHz, DMSO) δ 9.26 (s, 1H), 8.78 (s, 1H), 8.02 (t, *J* = 9.6 Hz, 1H), 7.48 (d, *J* = 8.6 Hz, 2H), 7.35 (d, *J* = 8.7 Hz, 2H), 7.32 – 7.25 (m, 1H), 6.82 (t, *J* = 8.3 Hz, 1H).; ^{13}C NMR (126 MHz, DMSO) δ 159.32, 157.43, 152.22, 149.24, 138.43, 129.13, 126.23, 120.09, 116.30, 116.22, 116.12, 116.04, 108.43, 108.36, 108.24, 108.17, 107.09, 106.85.; HRMS (EI) *m/z* calcd for $C_{13}H_{10}ClF_2N_2O$ [M+H] 283.0450, found 283.0486.

1-(4-chlorophenyl)-3-(3,4-difluorophenyl)urea (LMT-2349)

Isolated as a white solid (510 mg, 56% yield, Purification by column chromatography (20/1, petroleum ether/ethyl acetate); mp 254-256°C.; ^{1}H NMR (500 MHz, DMSO) δ 8.92 (s, 1H), 8.89 (s, 1H), 7.66 (dd, *J* = 11.5, 7.7 Hz, 1H), 7.48 (d, *J* = 8.4 Hz, 2H), 7.32 (d, *J* = 8.2 Hz, 2H), 7.32 (m, 1H), 7.12 (d, *J* = 8.0 Hz, 1H).;

^{13}C NMR (126 MHz, DMSO) δ 152.79, 150.54, 145.95, 145.88, 138.86, 137.16, 137.14, 137.08, 137.06, 129.05, 126.04, 120.34, 117.83, 117.71, 114.92, 107.78, 107.61.; HRMS (EI) *m/z* calcd for $C_{13}H_{10}ClF_2N_2O$ [M+H] 283.0450, found 283.0452.

1-(4-chlorophenyl)-3-(4-iodobenzyl)urea (LMT-2351)

Isolated as a white solid (600 mg, 50% yield, Purification by column chromatography (20/1, petroleum ether/ethyl acetate); mp 236-238°C.; ^1H NMR (500 MHz, DMSO) δ 8.75 (s, 1H), 7.68 (d, *J* = 8.2 Hz, 2H), 7.43 (d, *J* = 8.8 Hz, 2H), 7.26 (d, *J* = 8.8 Hz, 2H), 7.11 (d, *J* = 8.1 Hz, 2H), 6.69 (t, *J* = 5.9 Hz, 1H), 4.24 (d, *J* = 5.9 Hz, 2H).; ^{13}C NMR (126 MHz, DMSO) δ 155.41, 140.62, 139.76, 137.35, 129.84, 128.82, 124.93, 119.56, 92.73, 42.58.; HRMS (EI) *m/z* calcd for $C_{14}H_{13}ClIN_2O$ [M+H] 386.9761, found 386.9761.

1-([1,1'-biphenyl]-4-ylmethyl)-3-(4-chlorophenyl)urea (LMT-2352)

Isolated as a white solid (500 mg, 45% yield, Purification by column chromatography (20/1, petroleum ether/ethyl acetate); mp 222-224°C.; ^1H NMR (500 MHz, DMSO) δ 8.74 (s, 1H), 7.61 (t, *J* = 7.7 Hz, 4H), 7.43 (t, *J* = 8.4 Hz, 4H), 7.37 (d, *J* = 8.1 Hz, 2H), 7.33 (t, *J* = 7.3 Hz, 1H), 7.25 (d, *J* = 8.8 Hz, 2H), 6.70 (t, *J* = 5.9 Hz, 1H), 4.32 (d, *J* = 5.9 Hz, 2H).; ^{13}C NMR (126 MHz, DMSO) δ 155.46, 140.35, 139.90, 139.85, 139.05, 129.27, 128.84, 128.10, 127.67, 127.00, 126.93, 124.91, 119.54, 42.82.; HRMS (EI) *m/z* calcd for $C_{20}H_{18}ClN_2O$ [M+H] 337.1108, found 337.1101.

1-(4-aminobenzyl)-3-(4-chlorophenyl)urea (LMT-2353)

Isolated as a white solid (550 mg, 61% yield, Purification by column chromatography (20/1, petroleum ether/ethyl acetate); mp 272-274°C.; ^1H NMR (500 MHz, DMSO) δ 8.59 (s, 1H), 7.43 (d, *J* = 8.8 Hz, 2H), 7.25 (d, *J* = 8.8 Hz, 2H), 6.96 (d, *J* = 8.2 Hz, 2H), 6.52 (d, *J* = 8.2 Hz, 2H), 6.41 (t, *J* = 5.4 Hz, 1H), 4.97 (s, 2H), 4.10 (d, *J* = 5.5 Hz, 2H).; ^{13}C NMR (126 MHz, DMSO) δ 155.30, 147.96, 139.92, 128.84, 128.66, 127.12, 124.77, 119.41, 114.11, 42.99.; HRMS (EI) *m/z* calcd for $C_{14}H_{15}ClN_3O$ [M+H] 276.0904, found 276.0900.

1-(4-chlorophenyl)-3-(pyridin-4-ylmethyl)urea (LMT-2354)

Isolated as a white solid (600 mg, 70% yield, Purification by column chromatography (20/1, petroleum ether/ethyl acetate); mp 173-175°C.; ^1H NMR (500 MHz, DMSO) δ 8.89 (s, 1H), 8.50 (d, *J* = 5.8 Hz, 2H), 7.45 (d, *J* = 8.9 Hz, 2H), 7.28 (d, *J* = 5.8 Hz, 2H), 7.27 (d, *J* = 7.5 Hz, 2H), 6.80 (t, *J* = 5.9 Hz, 1H), 4.33 (d, *J* = 6.0 Hz, 2H).; ^{13}C NMR (126 MHz, DMSO) δ 155.54, 149.86, 139.73, 12909, 128.85, 125.07, 122.32, 120.19, 119.66, 42.19.; HRMS (EI) *m/z* calcd for $C_{13}H_{13}ClN_3O$ [M+H] 262.0747, found 262.0746.

1-(4-chlorophenyl)-3-((5-methylfuran-2-yl)methyl)urea (LMT-2355)

Isolated as a white solid (500 mg, 58% yield, Purification by column chromatography (20/1, petroleum ether/ethyl acetate); mp 175-177°C.; ^{1}H NMR (500 MHz, DMSO) δ 8.65 (s, 1H), 7.43 (d, *J* = 8.9 Hz, 2H), 7.26 (d, *J* = 8.9 Hz, 2H), 6.56 (t, *J* = 5.6 Hz, 1H), 6.11 (d, *J* = 2.8 Hz, 1H), 6.02 – 5.93 (m, 1H), 4.22 (d, *J* = 5.6 Hz, 2H), 2.23 (s, 3H).; ^{13}C NMR (126 MHz, DMSO) δ 155.08, 151.48, 151.00, 139.72, 128.87, 125.01, 119.52, 107.76, 106.71, 36.52, 13.65.; HRMS (EI) *m/z* calcd for $C_{13}H_{14}ClN_2O_2$ [M+H] 265.0744, found 265.0740.

1-(4-chlorophenyl)-3-((4-methylthiophen-2-yl)methyl) urea (LMT-2356)

Isolated as a white solid (560 mg, 61% yield, Purification by column chromatography (20/1, petroleum ether/ethyl acetate); mp 185-187°C.; ^{1}H NMR (500 MHz, DMSO) δ 8.71 (s, 1H), 7.43 (d, *J* = 8.9 Hz, 2H), 7.26 (d, *J* = 8.9 Hz, 2H), 6.93 (s, 1H), 6.79 (s, 1H), 6.68 (t, *J* = 5.8 Hz, 1H), 4.38 (d, *J* = 5.7 Hz, 2H), 2.15 (s, 3H).; ^{13}C NMR (126 MHz, DMSO) δ 155.14, 143.56, 139.71, 136.93, 128.84, 127.67, 125.00, 120.08, 119.57, 38.37, 15.79.; HRMS (EI) *m/z* calcd for $C_{13}H_{14}ClN_2OS$ [M+H] 281.0515, found 281.0508.

1-(4-chlorophenyl)-3-(4-((trifluoromethyl)thio)benzyl)urea (LMT-2359)

Isolated as a white solid (600 mg, 52% yield, Purification by column chromatography (20/1, petroleum ether/ethyl acetate); mp 191-193°C.; ^{1}H NMR (500 MHz, DMSO+ CD3OD) δ 7.67 (d, *J* = 8.1 Hz, 1H), 7.44 (dd, *J* = 8.2, 5.9 Hz, 2H), 7.24 (d, *J* = 8.9 Hz, 1H), 4.36 (s, 1H).; ^{13}C NMR (126 MHz, DMSO, MeOH) δ 155.67, 144.98, 139.82, 136.87, 131.47, 129.02, 125.39, 121.54, 119.87, 119.78, 42.76.; HRMS (EI) *m/z* calcd for $C_{15}H_{13}ClF_3N_2OS$ [M+H] 361.0389, found 361.0385.

1-(4-chlorophenyl)-3-(pyrimidin-5-yl) urea (LMT-2360)

Isolated as a white solid (550 mg, 68% yield, Purification by column chromatography (20/1, petroleum ether/ethyl acetate); mp 241-243°C.; ^{1}H NMR (500 MHz, DMSO) δ 9.15 (s, 1H), 9.04 (s, 1H), 8.91 (s, 2H), 8.82 (s, 1H), 7.51 (d, *J* = 8.9 Hz, 2H), 7.35 (d, *J* = 8.9 Hz, 2H).; ^{13}C NMR (126 MHz, DMSO) δ 152.68, 152.66, 152.37, 146.90, 138.54, 135.34, 129.04, 126.31, 120.51.; HRMS (EI) *m/z* calcd for $C_{11}H_{10}ClN_4O$ [M+H] 249.0543, found 249.0530.

Methyl 4-(3-(4-chlorophenyl)ureido)picolinate (LMT-2361)

Isolated as a white solid (659 mg, 66% yield, Purification by column chromatography (20/1, petroleum ether/ethyl acetate); mp 222-224°C.; ^{1}H NMR (500 MHz, DMSO) δ 9.46 (s, 1H), 9.10 (s, 1H), 8.48 (d, *J* = 5.5 Hz, 1H), 8.23 (d, *J* = 1.9 Hz, 1H), 7.59 (dd, *J* = 5.5, 2.1 Hz, 1H), 7.51 (d, *J* = 8.9 Hz, 2H), 7.36 (d, *J* = 8.9 Hz, 2H), 3.87 (s, 3H).; ^{13}C NMR (126 MHz, DMSO) δ 165.74, 152.33, 150.66, 148.59, 147.91, 138.30, 129.06, 126.54, 120.68, 115.45, 113.82, 52.74.; HRMS (EI) *m/z* calcd for $C_{14}H_{13}ClN_3O_3$ [M+H] 306.0645, found 306.0640.

Methyl 3-(3-(4-chlorophenyl)ureido)-4-methoxybenzoate (LMT-2362)

Isolated as a white solid (500 mg, 46% yield, Purification by column chromatography (20/1, petroleum ether/ethyl acetate); mp 196-198°C.; ^{1}H NMR (500 MHz, DMSO+ CD3OD) δ 8.80 (s, 1H), 7.61 (d, *J* = 9.9 Hz, 1H), 7.47 (d, *J* = 8.7 Hz, 2H), 7.28 (d, *J* = 8.7 Hz, 2H), 7.07 (d, *J* = 8.6 Hz, 1H), 3.94 (s, 3H), 3.80 (s, 3H).; ^{13}C NMR (126 MHz, DMSO+ CD3OD) δ 167.07, 152.96, 152.12, 139.29, 129.39, 129.21, 126.47, 124.76, 122.75, 120.21, 119.60, 110.86, 56.69, 52.35.; HRMS (EI) *m/z* calcd for $C_{16}H_{16}ClN_2O_4$ [M+H] 335.0799, found 335.0791.

1-(4-chlorophenyl)-3-(5-fluoropyridin-2-yl) urea (LMT-2363)

Isolated as a white solid (450 mg, 53% yield, Purification by column chromatography (20/1, petroleum ether/ethyl acetate); mp 204-206°C.; ^{1}H NMR (500 MHz, DMSO) δ 9.90 (s, 1H), 9.43 (s, 1H), 8.27 (s, 1H), 7.72 (qd, *J* = 9.1, 3.6 Hz, 2H), 7.53 (d, *J* = 8.8 Hz, 2H), 7.35 (d, *J* = 8.8 Hz, 2H).; ^{13}C NMR (126 MHz, DMSO) δ 156.35, 156.27, 154.32, 152.26, 149.55, 138.36, 134.81, 134.60, 129.09, 126.44, 126.35, 126.28, 120.52, 113.28.; HRMS (EI) *m/z* calcd for $C_{12}H_{10}ClFN_3O$ [M+H] 266.0496, found 266.0492.

1-(2-bromopyridin-4-yl)-3-(4-chlorophenyl) urea (LMT-2364)

Isolated as a white solid (504 mg, 58% yield, Purification by column chromatography (20/1, petroleum ether/ethyl acetate); mp 198-200°C.; ^{1}H NMR (500 MHz, DMSO) δ 9.29 (s, 1H), 8.16 (d, *J* = 5.6 Hz, 1H), 7.80 (d, *J* = 1.7 Hz, 1H), 7.50 (d, *J* = 8.9 Hz, 2H), 7.38 – 7.33 (m, 3H).; ^{13}C NMR (126 MHz, DMSO) δ 152.17, 150.82, 149.07, 142.34, 138.19, 129.08, 126.62, 120.68, 115.41, 112.65.; HRMS (EI) *m/z* calcd for $C_{12}H_{10}BrClN_3O$ [M+H] 325.9696, found 325.9691.

1-(4-bromonaphthalen-1-yl)-3-(4-chlorophenyl) urea (LMT-2365)

Isolated as a white solid (550 mg, 57% yield, Purification by column chromatography (20/1, petroleum ether/ethyl acetate); mp 276-278°C.; ^{1}H NMR (500 MHz, DMSO + CD_3OD) δ 8.16 (d, *J* = 7.6 Hz, 2H), 7.94 (d, *J* = 8.3 Hz, 1H), 7.80 (d, *J* = 8.3 Hz, 1H), 7.77 – 7.60 (m, 2H), 7.51 (d, *J* = 8.8 Hz, 2H), 7.31 (d, *J* = 8.8 Hz, 2H).; ^{13}C NMR (126 MHz, DMSO) δ 153.14, 139.07, 135.06, 131.88, 130.39, 129.20, 128.28, 127.81, 127.48, 127.21, 126.02, 122.65, 120.21, 118.68, 116.02,; HRMS (EI) *m/z* calcd for $C_{17}H_{13}BrClN_2O$ [M+H] 374.9900, found 374.9887.

1-(4-chlorophenyl)-3-(isoquinolin-4-yl) urea (LMT-2366)

Isolated as a white solid (600 mg, 57% yield, Purification by column chromatography (20/1, petroleum ether/ethyl acetate); mp 253-255°C.; ^{1}H NMR (500 MHz, DMSO) δ 9.22 (s, 1H), 9.06 (s, 1H), 8.99 (s, 1H),

8.86 (s, 1H), 8.13 (dd, *J* = 13.8, 8.4 Hz, 2H), 7.87 (t, *J* = 7.6 Hz, 1H), 7.72 (t, *J* = 7.5 Hz, 1H), 7.55 (d, *J* = 8.8 Hz, 2H), 7.36 (d, *J* = 8.8 Hz, 2H).; ^{13}C NMR (126 MHz, DMSO) δ 153.08, 147.68, 138.93, 135.64, 130.60, 129.71, 129.08, 128.99, 128.65, 128.27, 127.78, 125.93, 121.11, 120.13.; HRMS (EI) *m/z* calcd for $C_{16}H_{13}ClN_3O$ [M+H] 298.0747, found 298.0748.

1-(4-chlorophenyl)-3-(3,5-dichlorophenyl)urea (LMT-2367)

Isolated as a white solid (690 mg, 65% yield, Purification by column chromatography (20/1, petroleum ether/ethyl acetate); mp 231-233°C.; ^{1}H NMR (500 MHz, DMSO) δ 9.09 (s, 1H), 9.03 (s, 1H), 7.53 (d, *J* = 1.6 Hz, 2H), 7.48 (d, *J* = 8.8 Hz, 2H), 7.34 (d, *J* = 8.8 Hz, 2H), 7.16 (s, 1H).; ^{13}C NMR (126 MHz, DMSO) δ 152.50, 142.49, 138.54, 134.44, 129.02, 126.26, 121.38, 120.51, 116.77.; HRMS (EI) *m/z* calcd for $C_{13}H_{10}Cl_3N_2O$ [M+H] 314.9859, found 314.9848.

1-(4-chlorophenyl)-3-(3-fluoro-5-(trifluoromethyl)phenyl) urea (LMT-2368)

Isolated as a white solid (550 mg, 45% yield, Purification by column chromatography (20/1, petroleum ether/ethyl acetate); mp 182-184°C.; ^{1}H NMR (500 MHz, DMSO + CD_3OD) δ 7.65 (s, 1H), 7.59 (d, *J* = 11.2 Hz, 1H), 7.48 (d, *J* = 8.8 Hz, 2H), 7.30 (d, *J* = 8.9 Hz, 2H), 7.14 (d, *J* = 8.4 Hz,1H).; ^{13}C NMR (126 MHz, DMSO+ CD3OD) δ 164.01, 162.07, 152.84, 143.16, 143.07, 138.70, 120.81, 120.40, 111.16, 109.19, 108.98, 106.08, 106.04, 105.88, 105.85.; HRMS (EI) *m/z* calcd for $C_{14}H_{10}ClF_4N_2O$ [M+H] 333.0418, found 333.0406.

4-bromo-N-(4-chlorophenyl)-1H-imidazole-1-carboxamide (LMT-2369)

Isolated as a white solid (200 mg, 15% yield, Purification by column chromatography (20/1, petroleum ether/ethyl acetate); mp 169-171°C.; ^{1}H NMR (500 MHz, DMSO + CD_3OD) δ 7.61 (s, 1H), 7.46 (d, *J* = 8.4 Hz, 2H), 7.29 (d, *J* = 8.6 Hz, 2H), 7.21 (s, 1H).; ^{13}C NMR (126 MHz, DMSO+CD3OD) δ 154.56, 138.71, 136.37, 129.20, 126.74, 120.12, 116.18, 113.85.; HRMS (EI) *m/z* calcd for $C_{10}H_8BrClN_3O$ [M+H] 299.9539, found 299.9540.

N-(4-chlorophenyl)-2-cyano-1H-imidazole-1-carboxamide (LMT-2370)

Isolated as a white solid (210 mg, 17% yield, Purification by column chromatography (20/1, petroleum ether/ethyl acetate); mp 213-215°C.; ^{1}H NMR (500 MHz, DMSO + CD_3OD) δ 7.95 (s, 1H), 7.64 - 7.58 (m, 3H), 7.48 - 7.42 (m, 2H), 7.35 (d, *J* = 8.6 Hz, 2H).; ^{13}C NMR (126 MHz, DMSO+CD3OD) δ 146.60, 137.53, 131.48, 131.02, 130.50, 129.70, 129.27, 121.14, 115.75.; HRMS (EI) *m/z* calcd for $C_{11}H_8ClN_4O$ [M+H] 247.0387, found. 247.0374.

6-bromo-N-(4-chlorophenyl)-1H-benzo[d]imidazole-1-carboxamide (LMT-2371)

Isolated as a white solid (180 mg, 11% yield, Purification by column chromatography (20/1, petroleum ether/ethyl acetate); mp 164-166°C.; ^{1}H NMR (500 MHz, DMSO + CD_3OD) δ 8.22 (s, 1H), 7.75 (s, 1H), 7.52 (d, *J* = 8.5 Hz, 1H), 7.45 (d, *J* = 8.5 Hz, 2H), 7.32 (d, *J* = 8.5 Hz, 1H), 7.27 (d, *J* = 8.8 Hz, 2H).; ^{13}C NMR (126 MHz, DMSO) δ 152.81, 147.91, 145.20, 143.76, 143.60, 143.10, 142.92, 139.02, 137.14, 133.49, 131.57, 129.33, 129.09, 128.70, 127.88, 127.27, 125.97, 125.08, 122.98, 122.72, 122.68, 122.16, 120.29, 117.68, 117.51, 116.63, 116.46, 114.46.; HRMS (EI) *m/z* calcd for $C_{14}H_{10}BrClN_3O$ [M+H] 349.9696, found 349.9692.

Ethyl 5-(3-(4-chlorophenyl)ureido)benzofuran-2-carboxylate (LMT-2372)

Isolated as a white solid (440 mg, 35% yield, Purification by column chromatography (20/1, petroleum ether/ethyl acetate); mp 233-235°C.; ^{1}H NMR (500 MHz, DMSO + CD_3OD) δ 7.97 (s, 1H), 7.64 (s, 1H), 7.58 (d, *J* = 9.0 Hz, 1H), 7.47 (d, *J* = 8.8 Hz, 2H), 7.41 (dd, *J* = 9.0, 1.9 Hz, 1H), 7.28 (d, *J* = 8.8 Hz, 2H), 4.33 (q, *J* = 7.1 Hz, 2H), 1.32 (t, *J* = 7.1 Hz, 3H).; ^{13}C NMR (126 MHz, DMSO) δ 159.57, 153.45, 151.95, 146.56, 139.41, 136.53, 129.34, 127.85, 126.42, 121.02, 120.45, 114.81, 112.85, 112.31, 61.91, 14.64.; HRMS (EI) *m/z* calcd for $C_{18}H_{16}ClN_2O_4$ [M+H] 359.0799, found.

1-(4-chloronaphthalen-1-yl)-3-(4-chlorophenyl)urea (LMT-2373)

Isolated as a white solid (409 mg, 25% yield, Purification by column chromatography (20/1, petroleum ether/ethyl acetate); mp 310-312°C.; ^{1}H NMR (500 MHz, DMSO) δ 9.21 (s, 1H), 8.91 (s, 1H), 8.20 (s, 2H), 7.99 (dd, *J* = 8.2, 2.2 Hz, 1H), 7.73 (dd, *J* = 6.2, 2.6 Hz, 2H), 7.68 (dd, *J* = 8.3, 2.2 Hz, 1H), 7.54 (dd, *J* = 8.8, 2.1 Hz, 2H), 7.36 (dd, *J* = 8.8, 2.1 Hz, 2H).; ^{13}C NMR (126 MHz, DMSO) δ 153.07, 138.97, 134.29, 130.55, 129.09, 127.93, 127.56, 127.11, 126.62, 125.86, 125.16, 124.64, 122.57, 120.07, 118.11.; HRMS (EI) *m/z* calcd for $C_{17}H_{13}Cl_2N_2O$ [M+H] 331.0405, found 331.0408.

1-(benzo[d]oxazol-6-yl)-3-(4-chlorophenyl) urea (LMT-2374)

Isolated as a white solid (370 mg, 31% yield, Purification by column chromatography (20/1, petroleum ether/ethyl acetate); mp 239-241°C.; ^{1}H NMR (500 MHz, DMSO) δ 9.01 (s, 1H), 8.89 (s, 1H), 8.62 (s, 1H), 8.08 (s, 1H), 7.69 (d, *J* = 8.6 Hz, 1H), 7.50 (d, *J* = 7.0 Hz, 2H), 7.34 (d, *J* = 8.8 Hz, 2H), 7.26 (dd, *J* = 8.6, 1.9 Hz, 1H).; ^{13}C NMR (126 MHz, DMSO) δ 153.78, 152.84, 150.19, 138.92, 138.12, 134.70, 129.01, 125.84, 120.24, 120.20, 116.32, 100.93.; HRMS (EI) *m/z* calcd for $C_{14}H_{11}ClN_3O_2$ [M+H] 288.0540, found 288.0536.

1-(4-chlorophenyl)-3-(4-(4-methoxyphenyl)thiazol-2-yl)urea (LMT-2375)

Isolated as a white solid (190 mg, 13% yield, Purification by column chromatography (20/1, petroleum ether/ethyl acetate); mp 244-246°C.; ^{1}H NMR (500 MHz, DMSO + CD_3OD) δ 7.81 (d, *J* = 8.7 Hz, 2H), 7.52 (d, *J* = 8.8 Hz, 2H), 7.38 (s, 3H), 7.37 (d, *J* = 8.9 Hz, 2H), 6.98 (d, *J* = 8.8 Hz, 2H), 3.78 (s, 3H).; ^{13}C NMR

(126 MHz, DMSO) δ 159.38, 138.04, 129.27, 129.10, 127.61, 127.40, 126.82, 125.96, 120.69, 120.30, 114.52, 105.68, 55.61.; HRMS (EI) *m/z* calcd for $C_{17}H_{15}ClN_3O_2S$ [M+H] 360.0574, found 360.0569.

1-(4-chlorophenyl)-3-(4-nitronaphthalen-1-yl)urea (LMT-2376)

Isolated as a white solid (400 mg, 39% yield, Purification by column chromatography (20/1, petroleum ether/ethyl acetate); mp 248-250°C.; ^{1}H NMR (500 MHz, DMSO + CD_3OD) δ 8.59 (d, *J* = 8.6 Hz, 1H), 8.29 (d, *J* = 8.4 Hz, 1H), 7.76 (dt, *J* = 15.3, 7.4 Hz, 2H), 7.52 (d, *J* = 8.8 Hz, 2H), 7.44 (d, *J* = 8.8 Hz, 2H), 7.32 (d, *J* = 8.8 Hz, 2H), 7.26 (d, *J* = 8.8 Hz, 2H).; ^{13}C NMR (126 MHz, DMSO) δ 152.49, 141.69, 140.25, 138.51, 130.23, 129.34, 127.55, 126.89, 126.60, 126.14, 125.00, 123.88, 123.78, 122.46, 120.49, 113.51.; HRMS (EI) *m/z* calcd for $C_{17}H_{13}ClN_3O_3$ [M+H] 342.0645, found 342.0632.

4-bromo-N-(4-chlorophenyl)-3-cyano-1H-pyrazole-1-carboxamide (LMT-2377)

Isolated as a white solid (213 mg, 15% yield, Purification by column chromatography (20/1, petroleum ether/ethyl acetate); mp 239-241°C.; ^{1}H NMR (500 MHz, DMSO + CD_3OD) δ 8.17 (s, 1H), 7.44 (d, *J* = 8.7 Hz, 2H), 7.26 (d, *J* = 8.9 Hz, 2H).; ^{13}C NMR (126 MHz, DMSO+ CD3OD) δ 154.93, 138.98, 132.29, 129.41, 127.18, 120.44, 113.68, 98.60.; HRMS (EI) *m/z* calcd for $C_{11}H_7BrClN_4O$ [M+H] 324.9492, found 324.9497.

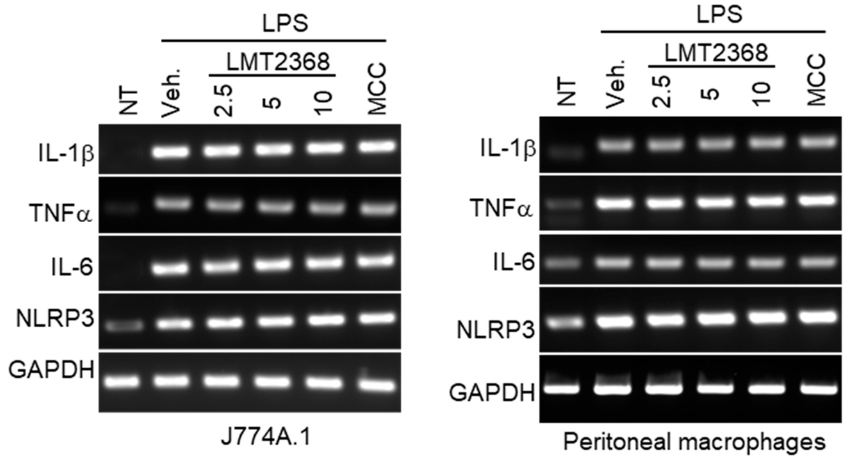

Supplementary Figure S1. LMT-2368 has no effect on LPS-stimulated gene expression. 774A.1 and peritoneal macrophages were treated with 1 μg/mL for 6 h in the presence of LMT2368. RNAs were isolated and subjected to RT-PCR for each gene.

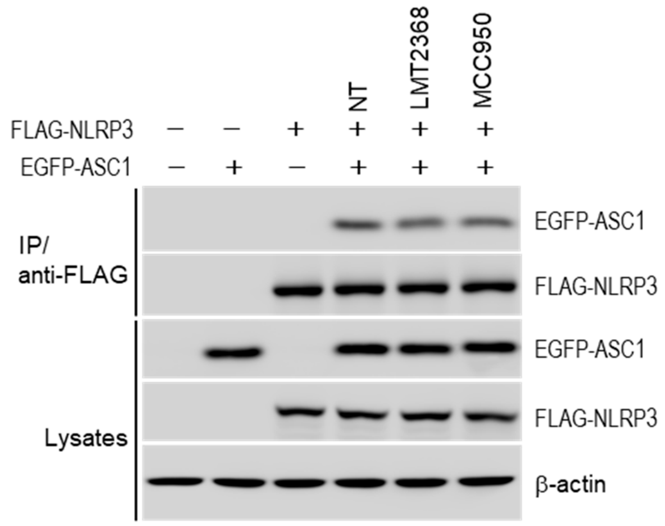

Supplementary Figure S2. LMT2368 has no effect on the interaction of NLRP3 and ASC. HEK293 cells were transfected with FLAG-tagged NLRP3 and EGFP-tagged ASC1, as well as an empty vector for the control group. Cells were incubated with 10 μM LMT2368 for 1 h and then lysed. The supernatants were incubated with anti-FLAG antibody-conjugated beads and then washed, resuspended in SDS sample buffer and boiled. The samples were analyzed by Western blotting with ASC or NLRP3 antibodies.

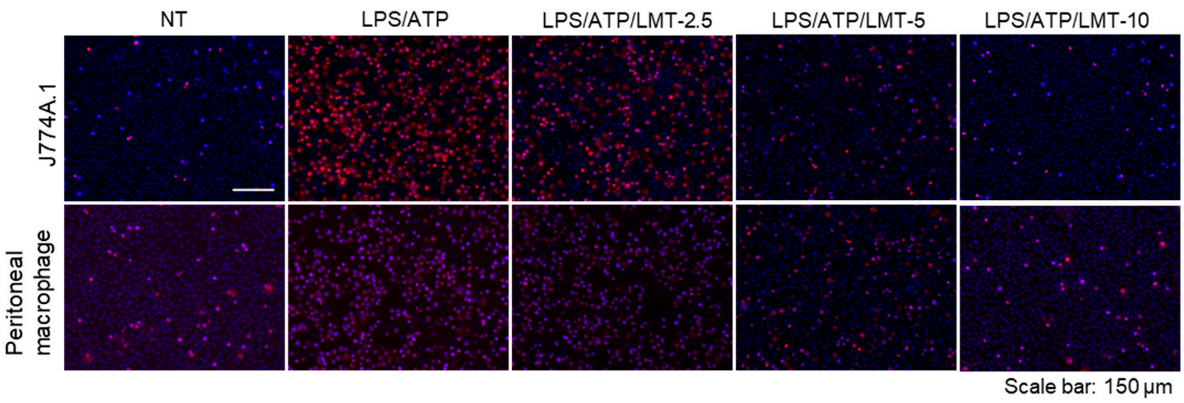

Supplementary Figure S3. LMT-2368 inhibits pyroptosis. LPS-stimulated cells were treated with LMT2368 prior to ATP application. The cells were incubated with PI and DAPI. Fluorescent images were obtained using EVOS M5000 Microscope imaging system.
